# Pathogens Inactivated by Low-Energy-Electron Irradiation Maintain Antigenic Properties and Induce Protective Immune Responses

**DOI:** 10.3390/v8110319

**Published:** 2016-11-23

**Authors:** Jasmin Fertey, Lea Bayer, Thomas Grunwald, Alexandra Pohl, Jana Beckmann, Gaby Gotzmann, Javier Portillo Casado, Jessy Schönfelder, Frank-Holm Rögner, Christiane Wetzel, Martin Thoma, Susanne M. Bailer, Ekkehard Hiller, Steffen Rupp, Sebastian Ulbert

**Affiliations:** 1 Fraunhofer-Institute for Cell Therapy and Immunology IZI, Perlickstrasse 1, 04103 Leipzig, Germany; jasmin.fertey@izi.fraunhofer.de (J.F.); lea.bayer@izi.fraunhofer.de (L.B.); thomas.grunwald@izi.fraunhofer.de (T.G.); 2 Fraunhofer-Institute for Organic Electronics, Electron Beam and Plasma Technology FEP, Winterbergstrasse 28, 01277 Dresden, Germany; pohl-alexandra@web.de (A.P.); jana.beckmann@fep.fraunhofer.de (J.B.); gaby.gotzmann@fep.fraunhofer.de (G.G.); javier.portillo@fep.fraunhofer.de (J.P.C.); jessy.schoenfelder@fep.fraunhofer.de (J.S.); frank-holm.roegner@fep.fraunhofer.de (F.-H.R.); christiane.wetzel@fep.fraunhofer.de (C.W.); 3 Fraunhofer Institute for Manufacturing Engineering and Automation IPA, Nobelstrasse 12, 70569 Stuttgart, Germany; martin.thoma@ipa.fraunhofer.de; 4 Fraunhofer Institute for Interfacial Engineering and Biotechnology IGB, Nobelstrasse 12, 70569 Stuttgart, Germany; susanne.bailer@igb.fraunhofer.de (S.M.B.); ekkehard.hiller@cvuas.bwl.de (E.H.); steffen.rupp@igb.fraunhofer.de (S.R.); 5 Institute for Interfacial Engineering and Plasma Technology IGVP, University of Stuttgart, 70569 Stuttgart, Germany

**Keywords:** pathogen inactivation, low-energy electron irradiation, vaccines, influenza A

## Abstract

Inactivated vaccines are commonly produced by incubating pathogens with chemicals such as formaldehyde or β-propiolactone. This is a time-consuming process, the inactivation efficiency displays high variability and extensive downstream procedures are often required. Moreover, application of chemicals alters the antigenic components of the viruses or bacteria, resulting in reduced antibody specificity and therefore stimulation of a less effective immune response. An alternative method for inactivation of pathogens is ionizing radiation. It acts very fast and predominantly damages nucleic acids, conserving most of the antigenic structures. However, currently used irradiation technologies (mostly gamma-rays and high energy electrons) require large and complex shielding constructions to protect the environment from radioactivity or X-rays generated during the process. This excludes them from direct integration into biological production facilities. Here, low-energy electron irradiation (LEEI) is presented as an alternative inactivation method for pathogens in liquid solutions. LEEI can be used in normal laboratories, including good manufacturing practice (GMP)- or high biosafety level (BSL)-environments, as only minor shielding is necessary. We show that LEEI efficiently inactivates different viruses (influenza A (H3N8), porcine reproductive and respiratory syndrome virus (PRRSV), equine herpesvirus 1 (EHV-1)) and bacteria (*Escherichia coli*) and maintains their antigenicity. Moreover, LEEI-inactivated influenza A viruses elicit protective immune responses in animals, as analyzed by virus neutralization assays and viral load determination upon challenge. These results have implications for novel ways of developing and manufacturing inactivated vaccines with improved efficacy.

## 1. Introduction

Vaccines consisting of inactivated pathogens are among the most widely used immunization strategies to fight infectious diseases of humans and animals. However, most of these vaccines still result from manufacturing procedures that have been developed decades ago and include limitations which need to be overcome by innovative methods. The inactivation is currently carried out by adding chemicals such as formaldehyde or β-propiolactone, a time-consuming method with varying reproducibility on inactivation levels and impacts on antigenic structures of the pathogen. The chemicals used are highly toxic and the material needs to be handled on elevated biosecurity levels in combination with the good manufacturing practice (GMP) guidelines during the whole period [1]. For many vaccine preparations the chemicals have to be removed, reverted into a nontoxic form, or diluted out after inactivation, which requires extensive downstream processing. Most importantly, the process often negatively impacts the efficiency of the resulting vaccines due to modification of the pathogen’s antigens by the inactivating chemical—e.g., via cross-linking of protein structures by formaldehyde [2,3]. As a consequence, critical antigens for inducing protective immune responses are affected by chemical inactivation and/or at worst the vaccine may even induce contrary effects such as hypersensitivity [4,5,6]. Hence, the development of novel inactivation procedures which act faster and better preserve the antigenic structures have the potential of generating more effective and affordable vaccines.

Alternative methods to chemical inactivation include radiation technologies. Ultraviolet light was shown to damage nucleic acids but also proteins, hence its application in vaccine development is challenging [7,8]. Most prominently, gamma- or high-energy electron radiation are being used for pathogen inactivation, but so far no human vaccine produced this way is available. Ionizing radiation has the advantages of working very quickly and, under conditions used for vaccine development, it preferentially damages nucleic acids rather than proteins, resulting in inactivated pathogens that are highly versatile to elicit effective immune responses [9,10,11]. Gamma radiation is emitted by a radioisotope and is able to efficiently penetrate liquids and solid materials. However, this method is complicated by the secondary radiation (Bremsstrahlung), which is induced by high energy radiation and cannot be controlled. As a consequence, damage to proteins via side-effects in the ionizing environment can increase [12,13]. In contrast, electron beam irradiation can be used as an adjustable on-off technology and provides a much more exact dosing option than gamma radiation. Therefore, shorter exposure time is needed and potential degradation of proteins is reduced. A limitation is that electron irradiation is less penetrating than gamma rays. The higher the energy for generating the electrons, the more penetrating the electrons are, however, when a high-energy (>1 MeV) electron beam is used, large amounts of X-rays are also generated. Therefore, similar to gamma-rays, irradiation facilities have to include complex shielding constructions for protection of staff and the environment. This excludes a direct integration of gamma radiation or high-energy technologies in biological production facilities. In contrast, low energy electron irradiation (LEEI, <500 keV), does not require complex shielding because the amount of X-rays generated is much lower. Until now, LEEI has mainly been used for sterilizing surfaces [14,15], since it is less penetrating than high energy electrons. As a consequence, liquid solutions must be present as thin liquid films (>1 mm) in order to be completely irradiated by LEEI. Due to the low secondary irradiation produced, LEEI can directly be integrated into standard laboratories, including GMP- and high biosafety level (BSL)-environments. Therefore, in principal, LEEI could be highly advantageous for the inactivation of pathogenic viruses or bacteria, e.g., in processes of vaccine manufacturing or other applications.

Here, we use LEEI for the first time to irradiate different pathogens in liquid solution and show that this technology can be used to completely inactivate viruses and bacteria while maintaining most of their antigenic structures. Moreover, after vaccination of mice with LEEI-inactivated influenza A virus we observe induction of efficient immune responses and protection after challenge with active virus, indicating a high potential for the prospective use of this inactivation technique for vaccine manufacturing.

## 2. Materials and Methods

### 2.1. Mice

Female BALB/c mice (6–8 weeks old) were obtained from Charles River (Sulzfeld, Germany). Groups of mice were kept in a pathogen-free environment in isolated ventilated cages.

All animal experiments were carried out in accordance with the EU Directive 2010/63/EU for animal experiments and were approved by local authorities (No.: TVV 07/15; DD24-5131/331/9).

### 2.2. Cells

Madin-Darby canine Kidney (MDCK) cells (ATCC, CCL-34) were used for all influenza A (H3N8) in vitro assays. Vero E6 cells (Vero C1008 (Vero 76, clone E6) ATCC, CRL-1586) were used for all equine herpesvirus 1 (EHV-1) in vitro assays. Cells were maintained in Dulbecco’s modified Eagle’s medium (DMEM) with l-glutamine (Thermo Fisher Scientific, Waltham, MSA, USA) containing 10% fetal bovine serum (FBS) and 1% Penicillin/Streptomycin (Thermo Fisher Scientific) at 37 °C with 5% CO_2_. For porcine reproductive and respiratory syndrome virus (PRRSV) in vitro assays, Marc145 cells (RIE 277 obtained from Collection of Cell Lines in Veterinary Medicine, FLI, Greifswald, Germany) were used and maintained in DMEM with l-glutamine (Thermo Fisher Scientific) containing 5% FBS, 5% non-essential amino acids (NEAA), 15 mM Hepes pH 7.5 and 1% Penicillin/Streptomycin (all from Thermo Fisher Scientific) at 37 °C with 5% CO_2_.

### 2.3. Pathogens

Influenza A/equine/Miami/2/63 (H3N8) was obtained from the virus collection at the FLI (Greifswald, Germany). After RNA extraction by QIAamp-Viral-RNA-Mini Kit (Qiagen, Hilden, Germany) and cDNA synthesis, the hemagglutinin (*HA*) gene was sequenced and identity of the strain was confirmed. For virus propagation and isolation, MDCK cells were inoculated with a multiplicity of infection (MOI) of ~0.1 in infection media (DMEM with Penicillin/Streptomycin and 0.5 μg/mL TPCK trypsin) and incubated for three days until a cytopathic effect (CPE) was visible. After centrifugation at 4600 rpm for 10 min at 4 °C, the cell debris were removed. The virus containing supernatant was stored in aliquots at −80 °C until inactivation.

To produce influenza A virus for enzyme-linked immunosorbent assay (ELISA), MDCK cells were inoculated with a MOI of 0.1 and incubated for three days until a CPE was visible. After removing the cell debris by centrifugation, supernatant was centrifuged through a discontinuous sucrose gradient ranging from 60% to 30% (*w/v*) in NTC-buffer (NaCl 100 mM, Tris-HCL pH 7.5, 20 mM, CaCl_2_, 50 mM) at 4 °C and 28,000 rpm for 3 h. The virus band was extracted and virus was pelleted at 4 °C and 30,000 rpm for 1 h. Pelleted virus was resuspended in NTC and stored in aliquots at −80 °C.

To produce influenza A virus for challenge experiments, fertilized eggs (clean eggs, Valo eggs, Germany) were infected with 100 µL of influenza A (H3N8) (1 × 10^3^ TCID50/mL) and incubated for 48 h. After refrigerating the eggs for 16 h at 4 °C, the allantoic fluid was extracted, diluted 1:2 with phosphate-buffered saline (PBS) and virus was concentrated by centrifugation at 28,000 rpm for 2 h at 4 °C through a 20% sucrose cushion in PBS. The virus was stored in aliquots at −80 °C.

Titers were measured by the tissue culture infectious dose-50 (TCID50) assay. To this end, 10,000 MDCK cells/well were plated on a 96-well plate the day before infection. The virus was added in 10-fold dilutions in infection media (DMEM with Penicillin/Streptomycin and 0.5 μg/mL TPCK trypsin) for each successive row of wells. The plates were stored in an incubator at 37 °C and 5% CO_2_. After five days, 70% ethanol was added to fix the cells and crystal-violet solution was added to stain the fixed cells. The plates were washed and the 50% infectious dose was calculated using the Reed–Muench method [16].

Equid herpesvirus 1 (EHV-1, obtained from Friedrich-Loeffler-Institut (FLI)) was cultivated in Vero E6 cells. For purification, cells were infected in serum free medium with a MOI of 0.01 and after incubation for 1 h at 37 °C medium was exchanged with DMEM containing 2% FCS. After the CPE was visible, the supernatant was removed from the cells and centrifuged at 1000 rpm for 10 min at 4 °C to remove the cell debris. The supernatant was filtered through a 0.45 µm filter and layered on a 20% sucrose cushion. After ultracentrifugation at 26,500 rpm for 2.5 h at 4 °C, the supernatant was removed, and the pelleted virus was resuspended in PBS. Aliquots were stored at −80 °C until used for irradiation.

PRRSV (European vaccine strain DV, MSD) was propagated on Marc145 cells according to [7]. For purification Marc-145 cells were infected in serum-free medium with a MOI of 0.1 and after incubation for 24 h at 37 °C medium was exchanged with fresh serum-free DMEM. After four days supernatant was removed from the cells and centrifuged at 4600 rpm for 10 min at 4 °C to remove the cell debris. The supernatant was layered on a 15% sucrose cushion. After ultracentrifugation at 28,000 rpm for 3 h at 4 °C the supernatant was removed, and the pelleted virus was resuspended in PBS. Aliquots were stored at −80 °C until used for irradiation.

*Escherichia coli* (strain DH5alpha harboring a puc18 plasmid for ampicillin resistance) was grown overnight in standard LB medium (Carl-Roth, Germany) at 37 °C and rotation at 200 rpm. The cells were washed twice in PBS and diluted to an OD_600_ of 3.0 in PBS before irradiation.

### 2.4. RNA Isolation and Bioanalyzer

RNA was isolated using the QIAamp-Viral-RNA-Mini Kit (Qiagen) according to the manufacturer’s instructions. For bioanalyzer tests the Agilent RNA 6000 Pico Kit (Agilent Technologies, Santa Clara, CA, USA) was used according to the manufacturer’s instructions. Samples were used directly or diluted with sterile water before analysis.

### 2.5. Quantitative Real-Time PCR

Transcripts were analyzed with the qPCR Kit: SuperScript^®^ III Platinum^®^ SYBR^®^ Green One-Step qRT-PCR Kit # 11736-051 (Invitrogen/Thermo Fisher Scientific) and the following Primers 5′-ACCGAGGTCGAAACGTACGT-3′ and 5′-CGCGATCTCGGCTTTGA-3′ flanking a 62 bp amplicon in the influenza A (H3N8) M1-open reading frame (ORF). Amplicons were quantified using a RNA-Oligo (Metabion, Planegg, Germany) as standard (ACCGAGGUCGAAACGUACGUUCUCUCUAUCGUACCAUCAGGCCCCCUCAAAGCCGAGAUCGCG) in dilutions from 10^8^ to 10^1^ copies/µL.

### 2.6. Pathogen Inactivation

#### 2.6.1. Irradiation

Samples were prepared by applying 230 µL of pathogen suspension into the center of a sterile 100 mm petridish (Primaria^TM^, Corning, NY, USA). The suspension was overlaid by a round OPP-foil resulting in a liquid film of even thickness of approx. 100 µm below the foil. Petri dishes were put on a cooled sample holder and covered with a layer of PET/PE-foil. The samples were then irradiated with the indicated doses with a 200 keV electron beam at 4 °C (KeVac System, Linac Technologies, Orsay, France (200 kV/5 mA)). Absorbed dose measurements were performed by applying a radiochromic dosimeter film (Risø B3 dosimeter, Risø High Dose Reference Laboratory, Roskilde, Denmark) into a petri dish as described above, followed by quantification of the color change at 554 nm (RisöScan-System, Risø High Dose Reference Laboratory) after each irradiation dose. The irradiated pathogens were stored in aliquots at −80 °C until subsequent analyses. The titer of the virus solution was determined prior and after inactivation. This was done by adding 10 µL/well of the respective irradiated solution in duplicates to fresh cells of the respective cell line seeded in 96-well plates. The plates were incubated for five days. To exclude any residual virus activity not visible after five days, the supernatants were passaged once to fresh cells. Inactivation was confirmed by performing the same cell culture tests with 20% of the total irradiated material. Inactivation of *E. coli* was confirmed by plating 10 µL of the irradiated solution on Luria broth (LB)-agar plates and incubation over night at 37 °C.

#### 2.6.2. Formaldehyde Inactivation

Influenza A (H3N8) containing cell culture supernatants were either incubated for 16 h at 4 °C with a final concentration of 0.1% (*v*/*v*) formaldehyde (F1635-25ML, Sigma-Aldrich, St. Louis, MO, USA) or for seven days at 37 °C with 0.05% (*v*/*v*) formaldehyde at 200 rpm. To mix the sample and to prevent virus aggregation, the solution was pipetted up and down once per day. After the procedure, the virus solution was dialyzed against PBS for 3 h at 4 °C. Dialysis tubes (ZelluTrans Roth E656.1, cutoff 3.5 kDa, width 19 mm, thickness 25 µm, Carl Roth, Karlsruhe, Germany) were soaked in PBS for at least 30 min and sealed with a clip-on top and bottom. Inactivation was confirmed as described above.

### 2.7. Hemagglutination Assay

To prepare erythrocytes for the hemagglutination assay, 1 mL of chicken blood was mixed with 49 mL of cold sterile PBS and centrifuged for 10 min at 800 rpm and 4 °C. The supernatant was discarded, the pelleted erythrocytes were subjected to three additional rounds of this procedure. Finally, cold PBS was added to obtain a 0.5% (*v*/*v*) chicken erythrocyte suspension and stored at 4 °C. Fifty microliters of inactivated virus preparations with a titer of 1 × 10^3^ TCID50/mL were serially two-fold-diluted in a 100 mL volume on a 96-well microtiter plate. The untreated virus stock was used, as positive control, and PBS served as the negative control. Fifty microliters of the chicken erythrocyte suspension in PBS was added to all wells and plates were incubated for 30 min at room temperature.

### 2.8. ELISA

Recognition of antigens before and after irradiation was determined by a direct enzyme-linked immunosorbent assay (ELISA). Briefly, wells of a 96-microwell plate were coated with dilutions of the respective pathogens in coating buffer (35 mM Na_2_HCO_3_/15 mM Na_2_CO_3_, pH 9.6) with a total volume of 100 μL per well and incubated at 4 °C overnight. Starting dilutions were 1:500 for EHV-1, 1:40 for PRRSV, influenza A and *E. coli*, with three subsequent 1:5 dilution steps. The wells were blocked with PBS containing 5% skim milk. Polyclonal sera were added and incubated for 2 h at room temperature. To detect influenza A (H3N8) antigens, serum from an influenza A infected pig (a kind gift from Hans-Joachim Selbitz, IDT Dessau) was used at a 1:100 dilution, to detect PRRSV antigens, serum from a PRRSV infected pig was used at a 1:100 dilution. To detect EHV-1 antigens, serum from an EHV-1 infected horse (a kind gift from Klaus Osterrieder, Institute for Virology, University Berlin) was used at a 1:1000 dilution. For *E. coli* antigens, a polyclonal peroxidase-conjugated rabbit anti-*E. coli* antibody (ABIN288146, antibodies-online) was used at a 1:300 dilution.

The levels of anti-influenza antibodies in sera were determined by coating purified influenza A (H3N8) (around 500 TCID_50_ per well). Sera from vaccinated mice were diluted in PBS, added to the ELISA wells, and incubated at 23 °C for 2 h.

After incubation with polyclonal sera, plates were washed with PBS containing 0.05% Tween 20, and incubated with a 1:5000 dilution of a peroxidase-conjugated rabbit anti-mouse immunoglobulin G antibody, goat anti-pig immunoglobulin G antibody, or goat anti-horse immunoglobulin G antibody, respectively (all from DAKO, Agilent Technologies, Santa Clara, CA, USA) at room temperature for 1 h. The plate was then washed three times, and TMB-ELISA substrate was used for color development (77248, BioLegend, San Diego, CA, USA) stopped by addition of 1 M H_2_SO_4_ after 30 min. The absorbance of reaction was then determined with a standard ELISA reader at 450 nm and reference wavelength at 520 nm.

### 2.9. Immunization and Challenge

Fifty microliters electron beam-irradiated (30 kGy) influenza A (H3N8) with a titer of approx. 3 × 10^4^ TCID_50_/mL was mixed with 50 µL 2% Alhydrogel (aluminium hydroxide gel adjuvant, aluminium content 10 mg/mL, Brenntag Nordic A/S, Denmark) per dose. The viruses inactivated with formaldehyde were formulated identically. Both the LEEI- and formaldehyde-treated viruses used for immunizations were derived from the same batch of cell culture supernatant with identical virus titers.

Groups of mice were vaccinated twice at four-week intervals by intramuscular administration. Control mice were not immunized. Serum samples were collected from blood of the animals one week before and on day 21 post immunization for ELISAs. Four weeks after prime immunization the animals received a booster immunization with the same vaccine or left untreated in the control group. Three weeks after booster immunization, the animals were bled again for collection of serum samples after the second immunization. Animals of both groups maintained a consistent weight prior to administration of the influenza challenge. Four weeks after the last vaccination, the mice were challenged intranasally with a sub-lethal dose (2 × 10^5^ TCID_50_) of influenza A (H3N8) after short inhalative isoflurane anesthesia, and the mice were scored daily for signs of infection. The clinical score was assessed by determination of the weight, the overall appearance, behaviour, and influence on the respiratory tract of the animals after influenza A (H3N8) infection. Mice were euthanized at indicated time-points with isoflurane pre-anesthesia followed by cervical dislocation. After lung explantation to 2 mL ice cold PBS, RNA was extracted from homogenated lung tissue. The homogenization of lungs was carried out with sterile single tube homogenizators (Miltenyi Biotec, Bergisch Gladbach, Germany). The homogenates were centrifuged at 2000× *g* for 5 min at 4 °C. The RNA was extracted from 140 µL of lung homogenate supernatant with the QIAamp-Viral-RNA-Mini Kit (Qiagen) according to the manufacturer’s instructions.

### 2.10. Virus Neutralization Assay

Blood was collected from the retrobulbar venous plexus of vaccinated (*n* = 5) or non-vaccinated (*n* = 5) mice three weeks after each vaccination under inhalative isoflurane anesthesia. The serum was stored in aliquots at −20 °C. For neutralization assays, 1 × 10^4^ MDCK cells were plated in each well of a 96-well plate. Serum was heat-inactivated for 30 min at 56 °C and diluted 1:10 with DMEM in the first row with subsequent 1:2 dilutions. A constant influenza A (H3N8) concentration of 2 × 10^2^ TCID_50_/well was used for each plate. Virus and serum were incubated at 37 °C for 1 h and then added to the 96-well plate containing MDCK cells and incubated for three days at 37 °C and 5% CO_2_. Crystal violet and 70% ethanol were added to visualize the results of the infection as described above for the TCID_50_ assay.

### 2.11. Statistical Analysis

Statistical analysis was performed using GraphPad Prism6 (GraphPad Software, Inc., La Jolla, CA, USA). ELISA and quantitative real-time PCR data were analyzed by one-way analysis of variance (ANOVA). Bonferroni post-test was used to compare LEEI with formaldehyde treated samples. Significance was set a priori at *p* ≤ 0.05. Each experiment was repeated in duplicate or triplicate. Irradiation was always performed in triplicate.

## 3. Results

### 3.1. Low-Energy Accelerated Electrons Efficiently Inactivate Pathogens and Lead to a Dose Dependent Fragmentation of Viral RNA

To analyze the suitability of LEEI for inactivation of pathogens in liquids, different viruses, and *E. coli* bacteria were irradiated in thin liquid suspensions. Different doses between 5 and 50 kGy were applied. Samples of pathogen containing liquids which were handled identically but without irradiation served as controls and are indicated as 0 kGy in the following. Upon irradiation, the material was analyzed for infectivity in cell culture-based infection assays for viruses or by plating on LB-agar for *E. coli*. *E. coli* was reproducibly inactivated at 5 kGy, the lowest dose applied in this study. The experiments showed no infectivity of PRRSV or EHV-1 after irradiation with 10 or 30 kGy (Table 1).

**Figure 1:**
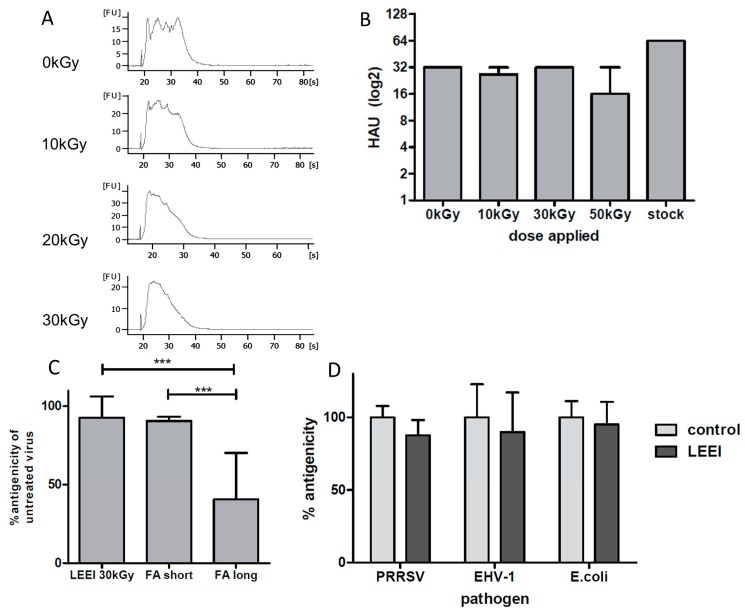
Characterization of the low-energy electron irradiation (LEEI) inactivated material. (**A**) Influenza A (H3N8) was LEEI-irradiated with the indicated doses (10 kGy, 20 kGy, and 30 kGy) or left non-irradiated (0 kGy) and RNA was isolated. Size and composition was analyzed by Agilent Bioanalyzer. The time scale on the *X*-axis represents the migration time in seconds, while FU represents the fluorescence intensity of the sample; (**B**) Median hemagglutination-units (HAU) of three independent experiments. HA-assay with LEEI-treated influenza A (H3N8)-material using chicken red blood cells in PBS. Non-irradiated (0 kGy) and irradiated (10 kGy, 30 kGy, and 50 kGy) samples were tested in triplicate. HA activity was measured as HAU and plotted as median with range; (**C**) Analysis of the antigen structure after inactivation by ELISA influenza A (H3N8) was inactivated by LEEI (30 kGy) or by adding formaldehyde either to a final concentration of 0.1% and incubation at 4 °C for 16 h (FA short), or to a final concentration of 0.05% followed by incubation at 37 °C for seven days (FA long). Untreated virus served as a positive control. Samples were coated on ELISA plates and probed with serum from an influenza A infected pig. Background was subtracted. Relative standard error of the mean (SEM) is indicated. *p*-values were determined by one-way ANOVA (*** *p* < 0.001); (**D**) LEEI inactivation is applicable to different pathogens. Porcine reproductive and respiratory syndrome virus (PRRSV) LEEI-inactivated with 30 kGy (LEEI), EHV-1 LEEI-inactivated with 10 kGy and *E. coli* LEEI-inactivated with 5 kGy and respective non-irradiated controls (control) were coated on ELISA plates and probed with serum from an PRRSV infected pig, serum from an EHV-1 infected horse, or a polyclonal anti-*E. coli* rabbit serum, respectively. Background was subtracted and ELISA results were normalized to the levels observed when active pathogens were used as antigen. Data from at least two independent experiments, each performed in triplicates are shown. Relative SEM is indicated.

Influenza A (H3N8) still showed residual activity after 10 kGy irradiation, while complete inactivation was reproducibly observed after application of 30 kGy or higher. For *E. coli*, the application of 5 kGy reduced the colony number of a starting culture with an OD_600_ of 3.0 (which corresponds to a cell number of around 2.4 × 10^9^ cells per mL) to 0. Different doses ranging from 5 kGy to 20 kGy were tested, however doses required for reducing the activity to 0, were not different when other starting titers were used (data not shown). For influenza A (H3N8) the integrity of the viral RNA was analyzed by a bioanalyzer. As demonstrated in Figure 1A, the RNA from the active virus is characterized by several distinct peaks, representing the intact RNA segments of the influenza A virus genome. Upon irradiation, the distinct peaks start to decline or even vanish, indicating the fragmentation of the nucleic acid in a dose-dependent fashion (Figure 1A). These data confirm earlier findings with other irradiation technologies and pathogens and suggest that LEEI inactivates by introducing strand breaks into nucleic acids [12,17].

### 3.2. Integrity of Antigens Is Maintained after LEEI for Influenza A and Other Pathogens

In order to test the integrity of the surface antigens of influenza A (H3N8), HA-assays were performed. The assay analyzes the ability of the HA-protein on the virus surface to agglutinate erythrocytes and is an established procedure to test for antigenicity of influenza viruses [2]. As demonstrated in Figure 1B, the non-irradiated control displayed an HA-activity of 32 HA-units (HAU). After LEEI-treatment this value remained almost constant and started to decline slightly after application of 50 kGy. To further investigate the integrity of the antigens of influenza A (H3N8), serum from infected animals containing polyclonal antibodies against influenza A was used in an ELISA. Upon complete inactivation with LEEI at 30 kGy, values for antigen integrities were around 90% of the non-irradiated control (Figure 1C). To analyze the antigenicity after different inactivation procedures and to validate the ELISA-based assay, we also treated influenza A viruses with formaldehyde (FA). Incubation was for 16 h (FA short) or for one week (FA long), as vaccine manufacturing inactivation times are usually extended to several days or weeks at 37 °C. The formaldehyde inactivated samples showed a conservation of antigenicity compared to the untreated sample of 90% after the short and 40% after the long incubation (Figure 1C). Short formaldehyde incubation displayed significantly better results than long incubation. In addition, LEEI performed statistically significantly better than the long treatment with formaldehyde (*p* < 0.001). This indicates that LEEI preserves antigenic structures better than formaldehyde under conditions used for vaccine manufacturing. Conservation of antigenic structures of the other pathogens was investigated using similar antibody-based assays with pathogen-specific polyclonal sera (Figure 1D). As shown for influenza A (H3N8), the antigenic structure of the other pathogens was also retained to a large extent after complete inactivation by LEEI: 87% for PRRSV, 89% for EHV-1, and 95% for *E. coli* compared to the respective non-irradiated control (Figure 1D). These results indicate that a high degree of antigen conservation during LEEI, at doses sufficient for complete inactivation, is a general feature of this inactivation method.

### 3.3. LEEI Inactivated Influenza A (H3N8) Viruses Induce Protective Immune Responses in Mice

**Figure 2:**
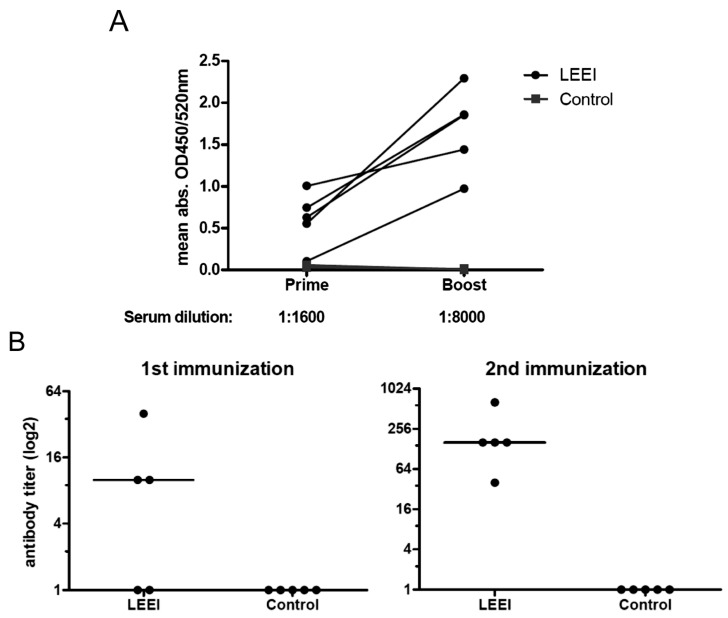
Antibody response in mice after vaccination (five animals per group). (**A**) purified influenza A (H3N8) from cell culture was coated on ELISA plates and probed with sera from vaccinated (LEEI) or non-vaccinated mice (control) after first vaccination (prime) and second vaccination (boost). Respective serum dilution is indicated below; (**B**) virus neutralizing antibody titers were analyzed by neutralization assays. Data points represent individual animals. Sera from vaccinated (LEEI) or non-vaccinated mice (control) after first immunization (left) and second immunization (right) were incubated with influenza A (H3N8) for 1 h and then added to fresh MDCK cells. After incubation for three days, cytopathic effect (CPE) was analyzed. CPE was absent at indicated antibody titers. Median of three independent experiments is indicated.

In order to investigate the suitability of LEEI-treated pathogens for a vaccine, inactivated influenza A (H3N8) viruses were used to immunize mice in a homologous prime-boost schedule. Aluminum hydroxide was used as an adjuvant as it is part of many available influenza vaccines. Influenza A specific antibodies were already detectable in the LEEI immunized mice after the first immunization (Figure 2A). The amount of antibodies increased after the second immunization, while no such antibodies were found in non-immunized animals of the control group. To analyze the amount of influenza A (H3N8) neutralizing antibodies, sera of mice were serially diluted, incubated with active virus, and transferred to MDCK cells. While no neutralizing antibodies were induced in the non-immunized animals, neutralizing antibodies were clearly detectable in three animals immunized with LEEI-inactivated viruses already after the prime, and in all mice of this group after the boost vaccination (Figure 2B).

**Figure 3:**
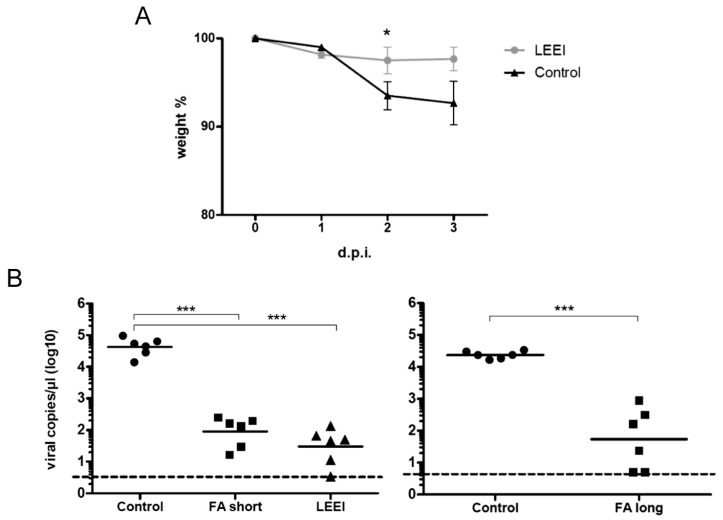
Challenge of vaccinated and unvaccinated mice. (**A**) weight loss in mice after intranasal challenge with a sub-lethal dose of influenza A (H3N8). Light grey circles: animals vaccinated with LEEI-inactivated material (LEEI), black triangles: non-vaccinated animals (control). *N* = 6 animals per group. SEMs are indicated. Unpaired *t*-test was used to calculate statistical differences (* *p* < 0.05); (**B**) determination of viral load in lung tissue of challenged mice 3 days post-infection (d.p.i.) by quantitative real-time PCR. Two independent experiments with identical experimental setups were conducted. Circles = non-vaccinated animals (control), rectangles = animals that received formaldehyde-inactivated material with two different treatments (left—short treatment with 0.1% for 16 h at 4 °C (FA short); right—long treatment with 0.05% for seven days at 37 °C (FA long)), triangles = animals that were immunized with LEEI-inactivated material (LEEI). *p*-value was determined by two-way ANOVA with bonferroni post-test (*** *p* < 0.001). The dashed line represents the detection limit of the assay.

To test whether the immunization with LEEI-treated influenza viruses can efficiently protect from infection with influenza A (H3N8), the immunized mice were intranasally challenged with a sub-lethal dose of virus [18]. First, three different infectious doses were tested in non-vaccinated animals (1 × 10^5^, 1 × 10^6^ and 1 × 10^7^ per animal) and the weight loss was analyzed daily (data not shown). According to the induced clinical score, we decided to use a sub-lethal dose of 2 × 10^5^ infectious influenza A (H3N8) per animal in the following challenge experiments. None of the vaccinated mice showed a significant clinical score after infection and all displayed a maximum of 2% average weight loss, while the average weight loss in the non-vaccinated control group was around 7% of body weight (Figure 3A).

Three days after infection mice were euthanized and RNA was isolated from lung tissue. Quantitative RT-PCR using primers that flank the M1-sequence of influenza A (H3N8) revealed that control mice had an average viral copy number of 5 × 10^4^/µL, while the LEEI-vaccinated mice displayed a statistically significant 982-fold reduction to 5.2 × 10/µL. (Figure 3B). For comparison, we also measured the protective capacity of the same amount of virus particles after short and long formaldehyde inactivation (Figure 1C). Upon immunization and challenge, the amount of viral genome copies in the lung was reduced by a factor of 382 (control compared to FA short) and 106 (control compared to FA long), respectively. Viral load reduction in both formaldehyde groups was statistically significant when compared to the non-vaccinated controls, but differences between the formaldehyde and LEEI groups were not (Figure 3B). Taken together, these data demonstrate that immunization of mice with LEEI-inactivated influenza A (H3N8) leads to an effective immune response and results in an efficient protection against infection.

## 4. Discussion

In the present study, we show that LEEI can be used to inactivate different pathogens in liquid solutions. Two single-stranded RNA viruses (influenza A (H3N8) and PRRSV), a double-stranded DNA virus (EHV-1), and a gram-negative bacterium (*E. coli*) were completely inactivated by LEEI. Upon irradiation, antigenic structures were almost indistinguishable from those of the active pathogens, as measured by binding of polyclonal antibodies and (for influenza A (H3N8)) by hemagglutination assays. The data suggest that LEEI-based inactivation is less damaging to native antibody binding sites than chemical inactivation with e.g. formaldehyde. The LEEI dose required to completely inactivate *E. coli* was lower than those for the different viruses, which is in line with data from other irradiation technologies. Higher resistance of viruses to ionizing radiation as compared to bacteria has been reported to be a general principle attributed to the relatively smaller genome sizes [12,19]. Ionizing radiation mainly targets the nucleic acids, due to their large G values compared to that of proteins or lipids. The G value represents the amount of radiolytic species that is produced per 100 eV of absorbed energy. Since the size of viral genomes is generally smaller than that of bacteria or eukaryotes, higher doses have to be applied for complete inactivation [20,21].

In accordance with previous data obtained with gamma- or high energy electron irradiation, LEEI-treatment of influenza A viruses leads to a dose dependent fragmentation of viral RNA, which is correlated to the inactivation of the virus [22,23]. The inactivated influenza A (H3N8) viruses were used to immunize mice and induced neutralizing antibodies in all animals. The vaccinated animals showed a significantly reduced viral load in the lungs upon challenge with active virus, which indicates that the immunization with LEEI-treated influenza A viruses mediates protection and therefore serves as a functional vaccine strategy. These data represent a proof-of-concept, but more work is required to validate the potential of this approach to replace existing methods for vaccine manufacturing. The reduction of viral load was higher in animals immunized with H3N8 inactivated by LEEI as compared to formaldehyde, but the differences were not statistically significant. Therefore, detailed analyses of immune responses resulting from the different inactivation methods need to be performed, in addition to studies on dosing, formulation, or the usage of alternative infection models.

Treatment of pathogens with ionizing radiation is a well-known alternative to the inactivation with toxic chemicals. Gamma radiation and high energy electron irradiation have been used to develop vaccine candidates against several different pathogens [11,19,24] and to sterilize food, medicinal instruments, or biological products [20,25,26,27]. LEEI has so far predominantly been employed for surface sterilization, as low-energy electrons have only a limited capability to penetrate materials and liquids. However, LEEI sources with an electron current output high enough for relevant dose application into thin liquid films, providing a very homogeneous irradiation profile, have been made available in recent years. To achieve the results presented here, irradiation was performed with electrons energized by 200 keV acceleration voltage, which is lower than high energy electron irradiation, where at least 1 MeV is used. This difference in energy has significant consequences for a potential application of irradiation in vaccine manufacturing: high-energy or radioactive (gamma radiation) power sources require large, complex, and expensive facilities to generate the energy and to shield the environment from ionizing radiation. In addition, due to the low dose rate of gamma irradiation, exposure times are higher than with electron irradiation and unwanted damage to antigenic structures is more likely to occur due to the non-controlled processes that are present under an ionized atmosphere [12,13,28,29]. On the other hand, LEEI-sources for irradiation of different materials are available and can easily be integrated and handled in normal laboratories, which in principal enables LEEI in GMP- and high BSL-facilities required for vaccine manufacturing. The irradiation time and the generation of X-rays are minimal; hence the effect of LEEI is predominantly direct and therefore very controllable and reproducible. Furthermore, there is no heat generated during inactivation, which also contributes to a better conservation of the antigens.

Previous studies have analyzed the sensitivity of various viruses to high energy electron irradiation [28,30,31]. The focus of most of these investigations was rather on reduction of infectivity than on complete inactivation, which is the most critical prerequisite for the generation of a vaccine. For influenza A viruses, a dose of 9.6 kGy for a 10,000-fold reduction of infectivity has previously been reported [28,32], whereas a roughly 1000-fold reduction was observed for influenza A with 10.4 kGy in the present study (Table 1). Different experimental setups in the two studies might account for the variation in inactivation rates, however, this finding also suggests that the effects of electrons on viral activity are similar at comparable doses, regardless whether high or low energy electrons are used.

In contrast to influenza A (H3N8), PRRSV, the second single-stranded RNA-virus analyzed in this study, was completely inactivated with a dose of 10.4 kGy at similar titers (Table 1). These differences might be attributed to different susceptibilities towards irradiation caused, for example, by distinct genome structures (one RNA strand in the case of PRRSV versus a segmented genome in influenza A), as observed previously for different viruses (reviewed in [28]) and for different genera of bacteria [33].

It was shown previously that the inactivation of microorganisms in liquids also depends on the protein concentration, the temperature, as well as the chemical composition of the solution. Preuss et al. reported that a higher dose was needed to inactivate the same amount of virus when the samples were frozen compared to liquid samples [31]. Especially in the area of food sterilization, several reports in the literature indicate that the matrix in which the organisms are present influences the reduction kinetics, resulting in differential reduction of infectivity [12,17,31,32]. In the study described here, influenza A (H3N8) viruses subjected to LEEI were present in clarified cell-culture supernatants, whereas PRRSV was purified and suspended in PBS buffer. The observed differences in inactivation doses highlight the importance of optimizing the irradiation to achieve the complete inactivation for every pathogen in its specific medium, independently.

In summary, these data represent the proof-of-concept for the usage of LEEI in pathogen inactivation for vaccine development. Several different viruses and even bacteria were shown to be sensitive to LEEI and to maintain their antigenic properties, and it is very likely that an even larger number of pathogens could be efficiently inactivated this way. This bears the potential to apply LEEI for the generation of a whole range of inactivated vaccines in human and veterinary medicine, provided that automated solutions for generating the thin liquid films required for effective inactivation are available. The limited penetration depth of low-energy electrons is a major challenge as, especially for vaccine production, large volumes of pathogen suspensions have to be processed. A continuous irradiation process might be the most suitable way to address this point. In order to transform LEEI into a process for industrial-scale vaccine manufacturing, developments of such solutions for a multi-liter-scale LEEI-based inactivation procedure are currently ongoing. However, this technology could also be very useful for other applications, such as diagnostics, where pathogenic microorganisms have to be inactivated fast and safely while maintaining their antigenic properties.

## Figures and Tables

**Table 1:** Absorbed dose and corresponding viral titers after low-energy electron irradiation (LEEI) treatment.

E-Beam Absorbed Dose (kGy ± SD)	Influenza A (H3N8) (log TCID50/mL ± SD)	PRRSV (log TCID50/mL ± SD)	EHV-1 (log TCID50/mL ± SD)
0	5.10 ± 0.6	5.42 ± 0.41	3.89 ± 0.15
10.4 ± 1.0)	2.32 ± 0.23)	0	0
29.9 ± 3.0)	0	0	0
50.2 ± 2.8)	0	0	ND

Absorbed dose (measured in kGy) and viral titers of at least two independent experiments (each performed in triplicates) are shown. Standard deviation (SD) is indicated in brackets. ND, not determined.
